# Identification of new breast cancer predisposition genes via whole exome sequencing

**DOI:** 10.1186/1897-4287-10-S2-A40

**Published:** 2012-04-12

**Authors:** MC Southey, DJ Park, F Lesueur, F Odefrey, T Nguyen-Dumont, F Hammet, SL Neuhausen, EM John, IL Andrulis, G Chenevix-Trench, L Baglietto, F Le Calvez-Kelm, M Pertesi, A Lonie, B Pope, O Sinilnikova, H Tsimiklis, GG Giles, JL Hopper, SV Tavtigian, DE Goldgar

**Affiliations:** 1 Genetic Epidemiology Lab, The University of Melbourne, Victoria, Australia; 2 International Agency for Research on Cancer, Lyon, France; 3 Dept of Population Sciences, Beckman Research Institute of City of Hope, Duarte, CA, USA; 4 Cancer Prevention Institute of California, Fremont, CA, USA; 5 Ontario Cancer Genetics Network, Cancer Care Ontario, Mount Sinai Hospital, Toronto, ON, Canada; 6 Queensland Institute of Medical Research, Queensland, Australia; 7 Centre for Cancer Epidemiology, The Cancer Council Victoria, Australia; 8 Victorian Life Sciences Computation Initiative (VLSCI), Victoria, Australia; 9 Unité Mixte de Génétique Constitutionnelle des Cancers Fréquents, Hospices Civils de Lyon/Centre Léon Bérard, Université de Lyon, France; 10 Centre for Molecular Environmental Genetic and Analytical Epidemiology, School of Population Health, The University of Melbourne, Victoria, Australia; 11 Department of Oncological Sciences, Huntsman Cancer Institute, University of Utah School of Medicine Salt Lake City, USA; 12 Department of Dermatology, University of Utah School of Medicine, Salt Lake City, Utah, USA

## 

The application of massively parallel sequencing (MPS) platforms has begun to revolutionize our understanding of the immense variation in the human genome and the complexity that can underlie genetic susceptibility to disease. The utility of exome capture MPS through the identification of genes for rare Mendelian disorders based on analysis of only a few individuals has been eloquently demonstrated. Common diseases such as breast cancer present substantially increased complexity in terms of locus, allelic and phenotypic heterogeneity, as well as complex relationships between genotype and phenotype (reduced penetrance, phenocopies etc.). With careful consideration of study design [1], thoughtful selection of families from our international resources (whole exome sequencing of two highly selected affected members of multiple-case breast cancer families), and a well-developed strategy (analytical pipeline) for distinguishing the few true breast cancer susceptibility genes from the many genes that have rare genetic variants that could plausibly alter protein function, we are advancing a large program of work aimed at identifying the majority of the “missing heritability” of breast cancer.

Our early findings demonstrate that:

1) despite very plausible biological roles, some genetic variants in some genes predicted to be damaging by SIFT and Polyphen2 do not appear to be associated with breast cancer risk [2].

2) application of our strategy can identify new breast cancer susceptibility genes. During the early conduct of this program, we identified a family with a protein truncating mutation in a gene involved in DNA repair. Follow-up has included mutation screening of:

a) youngest affected members of 250 multiple-case breast cancer families,

b) cases and controls participating in Australian population-based studies of breast cancer (ABCFS and MCCS)

c) cases and controls in an international population-based case-control-family resource (BCFR)

To date, these analyses have identified 6 families with frameshift or evolutionarily unlikely missense mutations in this gene. Features of these families include multiple-cases of early-onset female breast cancer with some potentially other interesting features such as early-onset male breast cancer and pancreatic cancer. These mutations have not been found in approximately 3000 unaffected population-based controls without a family history of breast cancer.

We are continuing to expand our dataset to include the exome sequences of further families and coordinating the follow-up of candidate genes using appropriate MPS platforms and as founding partners of a newly formed international consortium of breast cancer exome sequencing researchers.
